# Estimating the number and length of episodes in disability using a Markov chain approach

**DOI:** 10.1186/s12963-020-00217-0

**Published:** 2020-07-29

**Authors:** Christian Dudel, Mikko Myrskylä

**Affiliations:** 1 Laboratory of Population Health, Max Planck Institute for Demographic Research, Konrad-Zuse-Str. 1, Rostock, 18057 Germany; 2 Department of Social Policy, London School of Economics and Political Science, London, UK; 3 Population Research Unit, University of Helsinki, Helsinki, Finland

**Keywords:** Disability, Disability-free life expectancy, Activities of daily living, Number of episodes, Markov models, Markov chains with rewards, Matrix model, Matrix population models

## Abstract

**Background:**

Markov models are a key tool for calculating expected time spent in a state, such as active life expectancy and disabled life expectancy. In reality, individuals often enter and exit states recurrently, but standard analytical approaches are not able to describe this dynamic. We develop an analytical matrix approach to calculating the expected number and length of episodes spent in a state.

**Methods:**

The approach we propose is based on Markov chains with rewards. It allows us to identify the number of entries into a state and to calculate the average length of episodes as total time in a state divided by the number of entries. For sampling variance estimation, we employ the block bootstrap. Two case studies that are based on published literature illustrate how our methods can provide new insights into disability dynamics.

**Results:**

The first application uses a classic textbook example on prednisone treatment and liver functioning among liver cirrhosis patients. We replicate well-known results of no association between treatment and survival or recovery. Our analysis of the episodes of normal liver functioning delivers the new insight that the treatment reduced the likelihood of relapse and extended episodes of normal liver functioning. The second application assesses frailty and disability among elderly people. We replicate the prior finding that frail individuals have longer life expectancy in disability. As a novel finding, we document that frail individuals experience three times as many episodes of disability that were on average twice as long as the episodes of nonfrail individuals.

**Conclusions:**

We provide a simple analytical approach for calculating the number and length of episodes in Markov chain models. The results allow a description of the transition dynamics that goes beyond the results that can be obtained using standard tools for Markov chains. Empirical applications using published data illustrate how the new method is helpful in unraveling the dynamics of the modeled process.

## Background

Markov chains are routinely applied to model transitions between states. They are popular in part because they are easy to apply [1]. Given a set of probabilities or rates that describe the transitions between states, many useful quantities can be calculated with Markov chains, such as the expected time spent in a state [2–4]. In epidemiological and health research, Markov chains and other Markov models are, for example, often used to analyze active life expectancy and disability-free life expectancy [5–8], or life expectancy spent with or without specific conditions [9–12]. These health expectancies based on Markov models are in turn used as summary measures for assessing population health and the medical effectiveness and cost effectiveness of interventions [1, 13, 14].

While the time spent in a specific state derived from a Markov chain has many applications, this indicator may hide the dynamics of the underlying process. The expected time spent in a state gives us no information about how often a state is entered or left, e.g., whether it is entered only once for one long episode, or whether it is entered and left multiple times for many short episodes. While it can be safely assumed that some transitions occur only once—i.e., that the expected time in the state equals the length of the episode—in other cases a large number of repeated transitions to and from a state are possible. For example, this has been shown to be the case for frailty and disability [15, 16]. Up to now, mostly simulation methods were used for assessing this dynamic aspect of Markov processes [17], and analytical solutions have been limited to specific cases [18].

Using a discrete-time, homogeneous Markov chain with finite state space, we show how a general method for Markov chains with rewards presented by van Daalen and Caswell [19] and recently discussed in this journal by Caswell and Zarulli [20] can be used for the calculation of the expected number and length of episodes spent in a state. Modifying their method suffices to arrive at an easily applicable approach. We offer two empirical case studies to demonstrate the insights that can be gained by using our approach. Our first case study is a textbook example discussed by Andersen et al. that has been analyzed in numerous papers [21]. It is based on data from a clinical trial that assessed the survival and liver functioning of liver cirrhosis patients after treatment with prednisone, a steroid hormone. The second case study is based on recent work by Hardy and colleagues [15] and analyzes how the number and length of episodes of disability among older individuals vary depending on their levels of physical frailty.

To assess the statistical uncertainty of our calculations, we propose the block bootstrap as a method for estimation of the sampling variance when using longitudinal data. Estimating the sampling variance to conduct statistical inference and to, for instance, calculate confidence intervals has received relatively little attention in the literature. We use simulations to assess how reliable inference based on the block bootstrap is, and we compare it with other methods found in the literature.

We contribute to the literature in several ways. First, we extend the Markov chain toolbox by discussing a simple method to assess the dynamics captured by Markov chains. Second, extensive simulations included in the supplementary materials show that when using longitudinal data the block bootstrap is preferable compared to standard model-based bootstrap approaches. Third, we re-analyze datasets taken from the literature from a new perspective focusing on the number of episodes in good and bad health and the average length of these episodes. While we keep analyses simple, they highlight how our method could be used to shed new light on the dynamics of liver cirrhosis and prednisone treatment and disability and frailty. Fourth, the R code for the case studies and simulations is available online and readily implements the approach.

## Methods

### Preliminaries and basic notation

A Markov chain describes the transitions between a given set of states using transition probabilities. The set of possible states is called state space. For instance, in a classic illness-death model, the state space consists of three states: “healthy,” “ill,” (i.e., having a specific condition), and “dead.” A Markov chain evolves in discrete time and moves step by step from state to state; the step size can be chosen arbitrarily, and depending on the application, it could be 1 day, or 1 month, or 1 year. For instance, in an illness-death model with a step size of 1 month, at the beginning of the process, the state could be “healthy”; after 1 month, the state does not change and stays “healthy”; after another month, it changes to “ill”; and so on, until the state “dead” is reached, which is the so-called absorbing state which cannot be left. States which are not absorbing are called “transient.”

To formalize these ideas, our notation follows standard textbook treatments of Markov chains [2–4]. Let $Z_{t}\in \mathcal {S}$ denote the state a discrete-time Markov chain is in at $t=0,1,2,\dots $, for some finite state space $\mathcal {S}$ consisting of *m* states. The transitions between states are governed by transition probabilities Pr(*Z*_*t*+1_=*s*_*j*_|*Z*_*t*_=*s*_*i*_)=*p*_*ij*_ with $s_{i},s_{j}\in \mathcal {S}$, which capture the probability of moving from state *s*_*i*_ at time *t* to state *s*_*j*_ at time *t*+1. Transition probabilities only depend on the current state the Markov chain is in at time *t*, and not on any previous states at *t*−1,*t*−2, …. The Markov chain thus has the Markov property and is memoryless [2]. Throughout, we assume that the Markov chain is homogeneous, i.e., that it does not vary with *t*. Moreover, we assume that the Markov chain is absorbing, meaning that there are *q*>0 states that will definitely be reached and that will not be left. In the example of the illness-death model, the state “dead” is absorbing.

### Expected time in a state

Markov chains are usually analyzed in matrix notation. Transition probabilities are collected in the transition matrix **P**=[*p*_*ij*_], which is of dimension *m*×*m*, that is, the entry in the *i*th row and *j*th column of **P** is equal to Pr(*Z*_*t*+1_=*s*_*j*_|*Z*_*t*_=*s*_*i*_). Arranging transition probabilities in this way has been called a row-to-column orientation. Using the transition matrix, several quantities can be calculated [2–4]. The time spent in any non-absorbing (transient) state *s*_*j*_ starting from any transient state *s*_*i*_,*n*_*ij*_, can be calculated as

1 $$\begin{array}{@{}rcl@{}} \mathbf{N}=\left[n_{{ij}}\right]=\left(\mathbf{I}_{n}-\mathbf{U}\right)^{-1}, \end{array} $$

where **U** is a transition matrix that does not include absorbing states and thus only transition probabilities for moving between transient states; **I**_*n*_ is an identity matrix of dimension *n*×*n*, where *n*=*m*−*q* is the number of transient states; and the superscript −1 indicates the inverse matrix. The derivation can be found, for instance, in [2]. The row sums of **N** give the life expectancy conditional on starting in state *s*_*i*_. In the illness-death model from above, there are two transient states: healthy and ill. This means that the matrix **N** has two rows and two columns, and the row sums equal the life expectancy starting from the healthy state and the ill state, respectively.

### Markov chains with rewards

To estimate the expected number and length of episodes, we use Markov chains with rewards [22, 23]. An excellent introduction to Markov chains with rewards with application to population health was given by Caswell and Zarulli [20]. Generally, Markov chains with rewards are based on assigning rewards to transitions between states, and then allow for the calculation of the expected value of rewards. Rewards are collected in a matrix **R**=[*r*_*ij*_], where *r*_*ij*_ captures the reward for moving from state *s*_*i*_ to state *s*_*j*_. The expected number of rewards can be calculated as [19, 24]

2 $$\begin{array}{@{}rcl@{}} \mathbf{e}=\mathbf{N}\mathbf{Z}\left(\mathbf{P}\circ\mathbf{R}\right)\mathbf{1}_{m}. \end{array} $$

**1**_*m*_ is a column vector of length *m* with every entry equal to 1; **Z** equals (**I**_*n*_|**0**_*n*,*m*−*n*_), with **0**_*n*,*m*−*n*_ being a matrix of dimension *n*×(*m*−*n*) with all entries being equal to 0; and ∘ is used to denote the Hadamard product (i.e., the element-wise product). **e** is a vector of length *n* with the *i*th entry, *e*_*i*_, giving the expected number of rewards starting from transient state *s*_*i*_. Note that van Daalen and Caswell use a column-to-row orientation of the matrices, and not a row-to-column orientation like we do here. That means that Eq. (2) is a transposed version of the equations provided by van Daalen and Caswell [19, 24].

The intuition behind this is as follows. **N** captures the expected time spent in a state, which in the Markov chain is equivalent to the number of visits to a state. Given that we know how often a certain state *s*_*j*_ is visited, **P** includes the transition probabilities starting from this state, i.e., how probable it is that some other state *s*_*k*_ will be visited. If this state is visited, the reward is equal to *r*_*jk*_ captured by **R**. This means that Eq. (2) somewhat simplified amounts to checking for each state how often it will be visited; then checking how likely visits are to the other states; and what the reward is if such a transition happens. **Z** and **1**_*m*_ are used to calculate the overall sum of rewards.

### Expected number and length of episodes

Given Eq. (2), it turns out to be rather easy to calculate the number and length of episodes. Specifically, to calculate the expected number of episodes in a specific state *s*^∗^, we will set

3 $$\begin{array}{@{}rcl@{}} r_{{ij}}=\left\{\begin{array}{lll} 1 & & s_{i}\neq s^{*}, s_{j}=s^{*} \\ 0 & & \text{otherwise} \end{array}\right.. \end{array} $$

Moving from some state *s*_*i*_ to *s*^∗^ thus adds 1 to the value of rewards, i.e., the number of episodes. Other transitions, such as staying in state *s*^∗^ or moving from *s*^∗^ to some other state, do not add to the value of rewards. Given **R**, the expected number of episodes can be calculated using Eq. (2). The average length of episodes spent in *s*^∗^=*s*_*j*_ and starting from state *s*_*i*_,*a*_*ij*_ conditional on experiencing at least one episode can then be calculated using

4 $$\begin{array}{@{}rcl@{}} a_{{ij}}=n_{{ij}}/e_{i}. \end{array} $$

See the supplementary materials for a proof. As an example, consider again the illness-death model introduced above. If the entry of **R** corresponding to the transition from healthy to ill is set to one, then **e** will give the number of episodes in the ill state; more specifically, **e** will have two entries: one entry for the expected number of episodes of illness starting from the healthy state, and one entry for the expected number of episodes starting from the ill state. Combining this with entries from **N** like, for instance, the expected time in illness starting from the healthy state, allows to calculate *a*_*ij*_.

It is also possible to calculate the number of episodes in a subset of $\mathcal {S}$, i.e., the number of episodes in several states. This might be of interest if, for example, the state space includes several unhealthy states, and sickness episodes are being calculated. If $\mathcal {S^{*}}$ denotes this subset, the entries of **R** are set to

5 $$\begin{array}{@{}rcl@{}} r_{{ij}}=\left\{\begin{array}{lll} 1 & & s_{i} \not\in \mathcal{S}^{*}, s_{j}\in \mathcal{S}^{*} \\ 0 & & \text{otherwise} \end{array}\right.. \end{array} $$

Note that the interpretation of the results obtained using one of above variants in combination with Eq. (2) depends to some degree on the structure of the Markov chain. If, for instance, *s*^∗^ is a state that can only be left through an absorbing state—i.e., there can be at most one episode of that state—then the entries of **e** will give the probability of entering *s*^∗^ starting from *s*_*i*_. Otherwise, the approach essentially counts all transitions to state *s*^∗^ or subset of states $\mathcal {S}^{*}$. Replacing **R** in Eq. (2) with its transpose **R**^*T*^ will give the expected number of transitions out of state *s*^∗^, which can be used to calculate the number of recoveries.

### Sampling variance estimation

Estimating the sampling variance of **e** as given by Eq. (2) is not straightforward. The underlying issue is that while the sampling variance of transition probabilities can easily be calculated [25], this is not the case for highly non-linear functions of the transition matrix [26].

Both simulation-based methods and the bootstrap have been proposed as solutions for variance estimation for Markov chains. We adopt bootstrap approaches. A commonly used model-based bootstrap proceeds as follows [27]. Let *c*_*i*_ denote the observed number of individuals in state *s*_*i*_, and *c*_*ij*_ denotes the observed number of transitions from state *s*_*i*_ to *s*_*j*_. The maximum likelihood estimator of *p*_*ij*_ is *c*_*ij*_/*c*_*i*_. Bootstrap samples are constructed assuming a multinomial distribution with distribution $p_{i1},\dots,p_{{im}}$ and taking samples of size *c*_*i*_. For each bootstrap sample, the transition matrix is calculated and, based on this, the statistic of interest, such as **e**. The variance of **e** across samples is used as an estimator of its sampling variance.

This approach has the major shortcoming that it assumes that the data-generating process is (row-wise) i.i.d., i.e., that the data follows the Markov property. In applications with longitudinal data, the i.i.d. assumption is potentially invalid, as repeated transitions of the same individual are likely to be correlated.

Because of this shortcoming, we propose using a simple resampling procedure for longitudinal data based on the block bootstrap, as discussed by Cameron and Travedi [28] and Caswell [29], and sometimes applied in the context of multistate models [30]. All data belonging to the same individual is treated as one “block.” This could be, for instance, a sequence of health states: at *t*=0, the individual is healthy; at *t*=1, she is still healthy; at *t*=2, she has become sick, etc. Let the number of blocks be denoted by *B*, and the set of all blocks is $\mathcal {B}=\left \{b_{1},\dots,b_{B}\right \}$, with *b*_*k*_ being the block of data belonging to individual *k*. For each bootstrap replication, a new sample $\mathcal {B}^{*}$ is formed by sampling with replacement *B* blocks from $\mathcal {B}$. As in the case of the model-based bootstrap, the quantity of interest is calculated for each sample, and its variance across sample is used as an estimate of the sampling variance.

In the supplementary materials, we provide results of extensive simulations showing that the block bootstrap gives reliable variance estimates and that it performs considerably better than model-based bootstrap approaches. More specifically, the relative bias of the variance estimates of the block bootstrap is much smaller than the relative bias of model-based approaches, at least for most simulation variants. Because of this, we apply the block bootstrap in our first case study; for the second case study, we do not provide variance estimates due to data limitations.

## Results

### Case study 1: Prednisone treatment of liver cirrhosis

**Background**

Worldwide, liver cirrhosis is a leading cause of disability and death [31, 32]. Once established, liver cirrhosis cannot be cured and is accompanied by severe complications and a greatly increased risk of mortality [33]. Medication is prescribed to prevent or alleviate complications and to reduce mortality rates among patients with liver cirrhosis. Prednisone, a steroid hormone that was discovered in the 1950s, has been used in the treatment of cirrhosis complications. The results of early studies of this therapeutic approach were promising [34].

**Study population**

The first case study is based on a dataset used in a classic, textbook example discussed by Andersen et al. [21], which has been made available in the mstate package for R [35, 36]. The dataset consists of information on liver cirrhosis patients who entered a clinical trial in Copenhagen from 1962 to 1969. The main aim of the trial was to determine whether prednisone would increase the survival rates of these cirrhosis patients. The treatment group consisted of 251 individuals who received prednisone, and a control group of 237 patients who received a placebo. The survival rates and the prothrombin indexes were recorded for all of the patients. The prothrombin index is an indicator of the functioning of the liver, expressed as a percentage, with 100% indicating normal functioning. The patients were followed until 1974.

**Model**

**Fig. 1:**
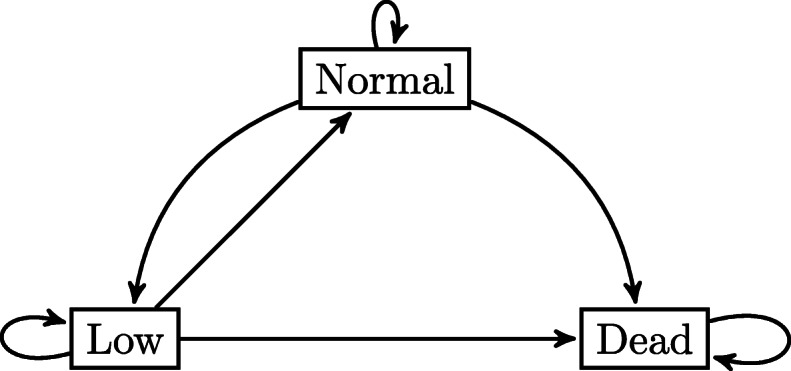
State space of the reversible illness-death model for the liver cirrhosis example: low prothrombin index value, normal prothrombin index value, dead

The model of the first case study, shown in Fig 1, is a reversible illness-death model, i.e., an illness-death model with recovery. The two states “normal” and “low,” which form the state space together with the absorbing state “dead,” are based on the prothrombin index and represent liver functioning. Prothrombin index values of less than 70% are classified as low functioning, while index values of 70% or higher are classified as normal functioning [35].

**Methods**

We calculated day-to-day transition probabilities in two steps. First, occurrence-exposure transition rates were estimated for both the treatment and the control groups, i.e., the number of transitions divided by exposure measured in days. Transition rates were collected in a matrix **B**, which was transformed into transition probabilities **P** through (**I**_*m*_−1/2**B**)(**I**_*m*_+1/2**B**)^−1^ [37]. Using these transition probabilities, the expected number of episodes of normal functioning and the expected length of episodes of normal functioning were calculated using the approach presented in this paper. In addition, the following quantities were estimated: the expected time with low functioning, the expected time with normal functioning, and the (partial) life expectancy. All of the calculations covered a 10-year period and were, for simplicity, based on the assumption that daily transition probabilities do not change over time or with age. Low functioning was the starting state. This means, for instance, that life expectancy for the control group shows the (partial) life expectancy for the next 10 years, given that liver functioning was abnormal. To calculate confidence intervals, the block bootstrap was used. Resampling proceeded at the level of patients, i.e., either all or none of the exposures and transitions of an individual was included in a bootstrap sample. 95% confidence intervals were based on the corresponding percentiles of the bootstrap distribution of the parameters resulting from 1000 bootstrap samples.

**Results**

The results are shown in Table 1. In line with well-known findings from the literature, we can see that the remaining life expectancy of the control and the treatment groups did not differ and that prednisone did not seem to prolong survival [21, 35]. But prednisone is shown to have affected how the patients’ remaining lifetime was spent: members of the treatment group spent an average of 3.7 years, or 62% of their remaining life expectancy, with normal liver functioning, compared to only 3.1 years, or 52% of remaining life expectancy, for the control group. Interestingly, taking prednisone was not found to increase the number of transitions from low to normal functioning: on average, members of the treatment group recovered from phases of low functioning as often as members of the placebo group. The increased lifetime with normal liver functioning found for the treatment group seems to have been solely due to longer episodes of normal functioning, conditional on having experienced such episodes. This result implies that prednisone neither prolonged survival nor increased the chances of recovery, but prevented relapse and helped maintain normal liver functioning after recovery, at least as measured through the surrogate outcome of the prothrombin index.

**Table 1:** Results on partial durations in states for case study 1 on liver cirrhosis and prednisone treatment

	Control	Treatment
Remaining life expectancy (years)	5.9	5.9
95% confidence interval	[5.6, 6.3]	[5.5, 6.3]
Expected time low functioning (years)	2.8	2.2
95% confidence interval	[2.6, 3.0]	[2.0, 2.5]
Expected time normal functioning (years)	3.1	3.7
95% confidence interval	[2.8, 3.4]	[3.4, 4.0]
Expected number of episodes (normal functioning)	0.8	0.8
95% confidence interval	[0.7, 0.9]	[0.7, 0.9]
Expected length of episodes (normal functioning, years)	3.9	4.5
95% confidence interval	[3.6, 4.2]	[4.3, 4.8]

### Case study 2: Physical frailty and disability in activities of daily living

**Background**

In the context of population aging, disabilities in activities of daily living (ADL) have been identified as a growing public health concern and have been intensively studied [38, 39]. Recovery from disability is not uncommon. Thus, individuals often experience multiple episodes of disability [40]. Based on the strong evidence that physical frailty is a major driver of disability [41], we can expect that frail individuals will experience more and longer episodes of disability than nonfrail individuals.

**Study population**

Our second case study uses the results of an analysis by Hardy et al. of data collected in New Haven, CT, USA, from 1998 to 2004 [15]. The dataset captures the disability status of 754 study participants aged 70 and older, with a median follow-up period of 60 months. The participants’ disability levels were assessed during monthly telephone interviews, in which they were asked about their levels of independent functioning in four activities of daily living: bathing, dressing, walking, and transferring. The participants’ physical frailty levels were measured based on whether their rapid gait test score was higher than 10 s. Each individual’s disability status was assessed at baseline and then every 18 months.

**Model**

**Fig. 2:**
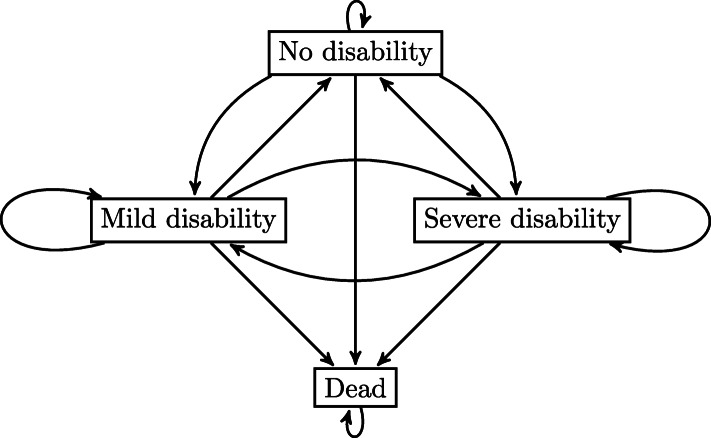
State space of the model of Hardy et al. [15]

Hardy et al. distinguished between four states, shown in Fig. 2: “no disability,” “mild disability,” “severe disability,” and the absorbing state “dead” [15]. The participants were classified as having mild disability if they were disabled in one or two ADLs, and they were classified as having severe disability if they were disabled in three or four ADLs. Hardy et al. published counts of transition events and exposures, which allow for the calculation of monthly transition rates between the states. The transition rates were transformed into monthly transition probabilities, as described for the first case study. These transition probabilities were used to calculate the expected time spent in each state, the expected number of episodes of disability, and the expected episode length. For the number of episodes, we counted the transitions to disability, i.e., we counted both the transitions to mild or severe disability from the no disability state. All of these measures were calculated over a period of 60 months (i.e., the median follow-up period) and differentiated between frail and nonfrail states. For this case study, the block bootstrap cannot be applied, as it requires patient-level data and information on individual trajectories, while we only have access to the aggregated transition rates. Because of this, we refrain from resampling and do not report confidence intervals or standard errors.

**Results**

Table 2 shows the results for the expected time in each of the states, the expected number of disability episodes, and the average length of disability episodes. All of these results are conditional on starting in the no disability state at the beginning of the 60-month period under consideration. This means, for instance, that a nonfrail and non-disabled older individual over a period of 60 months can expect to spend roughly 53 months, or 89% of the period under consideration, in the no disability state. For a frail individual, on the other hand, only 36 months, or 60% of the period under consideration, were lived without disability. When we added together the time spent in mild and severe disability, we found that frail individuals spent a considerably longer average period time in disability (13.6 months) than nonfrail individuals (2.2 months). This difference was partly driven by the expected number of disability episodes experienced during the 60-month period. On average, frail individuals experienced three times as many episodes of disability as nonfrail individuals. In addition, the disability episodes were twice as long for frail than for nonfrail older individuals.

**Table 2:** Results on partial durations in states for case study 2 based on transition rates taken from [15]

	Nonfrail	Frail
Expected time non-disabled (months)	53.4	36.2
Expected time mild disability (months)	1.8	9.5
Expected time severe disability (months)	0.4	4.0
Expected number of disability episodes	0.7	2.2
Expected length of disability episodes	3.1	6.2

## Discussion

The two empirical case studies presented here highlight the usefulness of assessing the expected number and length of episodes. The first case study found that prednisone treatment neither prolonged survival nor increased the probability of recovery, but that it reduced the likelihood of relapse and extended episodes of normal liver functioning, thus showing its efficacy compared to a placebo treatment. The second case study showed that there were considerable differences in both the number and the length of disability episodes between frail and nonfrail elderly individuals. Both examples show that the new perspective presented in this paper is helpful in unraveling the dynamics of the modeled process and that it allows for a detailed assessment of differences between groups. The results of our approach could also be used to enrich analyses of cost effectiveness of a given treatment like prednisone by, for instance, assessing the costs of increasing the number of episodes in good health.

While our analyses show the usefulness of the method discussed in this paper, they serve mostly as examples, and their validity might be somewhat limited by, for instance, not controlling for important stratifying variables. The second example might also suffer from its reliance on monthly transition data, which could be too coarse to give reliable estimates of the number of episodes [42]. While the use of such data might also affect estimates of the expected time spent in a state [43], there is evidence that for many applications, the estimation of the time spent in a state is relatively insensitive to the time unit used [44]. Thus, calculating the number and length of episodes might require additional care.

More generally, for the approach presented in this paper to yield valid results, valid estimates of transition probabilities are required, and the Markov chain must be an appropriate representation of the process that is being modeled. The assumptions about, for example, the Markov property or the homogeneity of the Markov chain, must accurately reflect this process. While many potential issues associated with Markov process modeling have been tackled in the literature, and can often be addressed when estimating transition rates or probabilities, our method does not account for these issues specifically [11, 45–47]. Our simulation results presented in the supplementary materials show that in case of correlated longitudinal data variance estimates can be improved by using a block bootstrap. Not taking into account the correlation structure can lead to substantially overestimating the sampling variance, although its data demands are higher than for simpler bootstrap methods. Irrespective of the bootstrap variant, resampling yields rather unreliable results for small samples, though.

Our approach can be applied to arbitrary complex state spaces. This feature could, for instance, be useful when transition probabilities are age-dependent (or time-dependent). In such a case, the state space could be expanded to include age: e.g., “aged 50 and non-disabled,” “aged 51 and non-disabled,” and so on. In this case, the values in the matrix of rewards **R** that relate to transitions from “non-disabled” to “disabled” are set to 1, e.g., for the transition from “aged 50 and non-disabled” to “aged 51 and disabled”. Furthermore, our method can be applied not only to cases in which the number and length of episodes are themselves of interest, but also to the assessment of the fit of the model: in cases in which a Markov approach is used and a priori knowledge about the process and the number of episodes is available, the results can be compared to the predictions of the model.

## Conclusions

In this paper, we built on previous work by van Daalen and Caswell and presented a method based on Markov chains with rewards that can be used to calculate the expected number and length of episodes in a state for a discrete-time, finite-state Markov chain [19]. Variance estimation can proceed using the block bootstrap. To illustrate how easily this approach can be applied, we presented two case studies: an illness-death model of liver functioning without recovery, and a more complex model of disability. In both cases, our approach yielded insights into the modeled process that went beyond the results that could be obtained using standard tools for Markov chains.

## Supplementary information

**Additional file 1** This file contains a proof of Eq. (4), a description of the simulations we conducted to assess the performance of the bootstrap approaches, and the results of the simulations.

**Additional file 2** We provide twelve files of R code which allow to replicate our examples and simulations, and an additional file containing a brief description of the individual files of code.

## Data Availability

All R code needed to replicate the results is available in supplementary files. The data of the first case study is available as part of the R package mstate which is available via CRAN: https://cran.r-project.org/web/packages/mstate/index.html. The data of the second case study was directly taken from a table given in the paper by Hardy and colleagues [15] and is reproduced in our R code.

## Abbreviations

ADL: Activities of daily living
