# Supplementary material for: Estimating the number and length of episodes in disability using a Markov chain approach
Source: Popul Health Metr. 2020 Jul 29;18:15. doi: 10.1186/s12963-020-00217-0 (PMC7389377; doi:10.1186/s12963-020-00217-0)
Supplement: Supplementary file 1 — Additional file 1 This file contains a proof of Eq. (4), a description of the simulations we conducted to assess the performance of the bootstrap approaches, and the results of the simulations. [file 12963_2020_217_MOESM1_ESM.pdf]

Supplementary materials for “Estimating the expected number and length of episodes using Markov chains with rewards”

Christian Dudel*[1] and Mikko Myrskylä†[1,2,3]

[1]*Laboratory of Population Health, Max Planck Institute for Demographic Research*
[2]*Department of Social Policy, London School of Economics and Political Science*
[3]*Population Research Unit, University of Helsinki*

*Corresponding author; address: Max Planck Institute for Demographic Research, Konrad-Zuse-Str. 1, 18057 Rostock, Germany; email: dudel@demogr.mpg.de; phone: +49 381 2081221; fax: +49 381 2081521
†Email: myrskyla@demogr.mpg.de

1 Proof of equation (4)

As was pointed out by Frishman[1], the ratio of the expectations of two random variables is not necessarily equal to the expectation of the ratio of the variables. Formally,

$$\mathrm{E}\left(\frac{Y}{X}\right) = \frac{\mathrm{E}(Y)}{\mathrm{E}(X)} - \frac{\mathrm{Cov}(X, \frac{Y}{X})}{\mathrm{E}(X)},$$

where X and Y denote the random variables. In the manuscript in equation (4), we have

$$\begin{aligned} \mathrm{E}(X) &= n_{ij} \\ \mathrm{E}(Y) &= e_j, \end{aligned}$$

which thus does not necessarily equal $a_{ij} = \mathrm{E}(Y/X)$. Here, we show that in our case $\mathrm{E}(Y/X) = \mathrm{E}(Y)/\mathrm{E}(X)$ holds. For this, we use the Notation of Frishman. Let Y denote the time spent in a state, and X captures the number of episodes.

First, note that $\mathrm{E}(Y/X)$ is not defined, as X can equal 0; in a sense, the average length of episodes is not defined if there are no episodes. Instead, as described in the paper we use $\mathrm{E}(Y/X|X > 0)$; i.e., we condition on observing at least one episode. This can also be done for the right-hand side of Frishman's equation:

$$\mathrm{E}\left(\frac{Y}{X}|X > 0\right) = \frac{\mathrm{E}(Y|X > 0)}{\mathrm{E}(X|X > 0)} - \frac{\mathrm{Cov}(X, \frac{Y}{X}|X > 0)}{\mathrm{E}(X|X > 0)}.$$

Next we argue that $\mathrm{Cov}(X, \frac{Y}{X}|X > 0)$ equals 0 due to the Markov property. Consider, for instance, a simple illness-death model. Every time an individual transitions from healthy to ill the length of the time she stays ill is not determined by previous periods of illness. Because of this, the second term on the right-hand side of the equation above equals zero.

Finally, we show that

$$\frac{\mathrm{E}(Y|X > 0)}{\mathrm{E}(X|X > 0)} = \frac{\mathrm{E}(Y)}{\mathrm{E}(X)},$$

and thus that equation (4) is correct. Moving from $\mathrm{E}(Y)$ to $\mathrm{E}(Y|X > 0)$ and from $\mathrm{E}(X)$ to $\mathrm{E}(X|X > 0)$, respectively, only requires multiplication with $1/\mathrm{Pr}(X > 0)$. First, $\mathrm{E}(Y) = \sum_{y=0}^{\infty} Pr(Y = y)y = \sum_{y=1}^{\infty} Pr(Y = y)y$. Second, as summation is running from $y = 1$ to higher values, $Pr(Y = y)/\mathrm{Pr}(X > 0) = \mathrm{Pr}(Y = y|X > 0)$, as $\mathrm{Pr}(Y = y) = \mathrm{Pr}(Y = y, X > 0)$

for $y \geq 1$. This means that

$$\begin{aligned} 1/\Pr(X>0)\mathrm{E}(Y) &= \sum_{y=1}^{\infty} Pr(Y=y)/\Pr(X>0)y \\ &= \sum_{y=1}^{\infty} \Pr(Y=y|X>0)y \\ &= \mathrm{E}(Y|X>0). \end{aligned}$$

The same argument holds for $\mathrm{E}(X)$ and $\mathrm{E}(X|X>0)$. Thus,

$$\frac{\mathrm{E}(Y|X>0)}{\mathrm{E}(X|X>0)} = \frac{1/\Pr(X>0)\mathrm{E}(Y)}{1/\Pr(X>0)\mathrm{E}(X)} = \frac{\mathrm{E}(Y)}{\mathrm{E}(X)}.$$

As part of the R code we provide simulations and a numerical example which lend further support to our theoretical result. Specifically, we provide two simulation setups. The first setup is similar in structure to the first case study we present in the main paper, while the second setup uses the state space of the second case study. For each setup, we generate 250 thousand sampling paths, and calculate the average length of episodes based on this data. Comparing results to the analytical solution provided by equation (4) shows that estimates match and that differences are negligible. The numerical example included in the R code is constructed in such a way that the average length of episodes in the state of interest is equal 1. Equation (4) also provides correct results in this case.

2 Bootstrap simulation setup

To assess whether the block bootstrap or the model-based bootstrap perform better in settings with and without correlated transitions, we conducted a small simulation study. We simulate transition data for large populations with different degrees of correlation between transitions, draw samples of different sizes, and apply both the model-based and the block bootstrap. The resulting estimates of the variance are compared to the true values.

More specifically, we created transition data for three populations of $250,000$ individuals each. For each individual, between one and nine transitions are generated, using a state space as shown in Figure 1, with two transient and one absorbing state. The initial state an individual is in is drawn from the two transient states, each with the same probability. In the first population, all individuals follow the same transition probabilities, and transitions are drawn i.i.d. given the starting state s_i.

For the second and the third population, different data-generating processes are used and unobserved heterogeneity is introduced. Both populations consist of two groups with

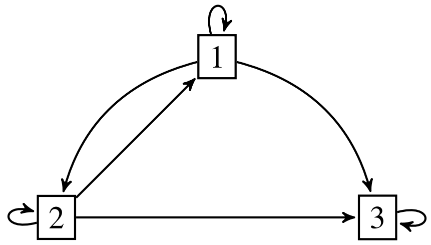

Figure 1: State space of the simulated data.

different transition probabilities, with the first group being more likely to stay in, or move to, state 1, whereas the second group is more likely to be observed in state 2. Group membership is drawn randomly. Except for simulating and generating the data of the populations, group membership is assumed to be unknown and not accounted for when applying Markov chain methods. This unobserved heterogeneity amounts to a non-i.i.d. setting and induces serial correlation of states. For the first non-i.i.d. population groups are more similar than for the second population, which shows strong heterogeneity. The specific transition matrices used for all three populations are given in the supplementary materials.

From both the i.i.d. and the non-i.i.d. populations $5,000$ samples of 50, 100, 250, 500, and $1,000$ individuals are drawn, always using all transitions per individual. For each of the resulting $75,000$ samples, transition probabilities are estimated using the ML estimator for p_{ij}, and based on the resulting transition matrix we calculate estimates of the expected time spent in state 1, n_1, as well as the expected number of episodes in state 1, e_1. We estimate the sampling variance of $\hat{n}_1$ and $\hat{e}_1$ based on both the model-based bootstrap as well as the block bootstrap, using $5,000$ bootstrap replications for each. Estimates for n_2 and e_2 will not be reported, as they are highly correlated with e_1 and n_1, and thus the findings are very similar to those for e_1 and n_1.

For each setting (i.i.d./non-i.i.d. population, sample size) we assess the performance of the bootstrap methods by calculating their relative bias, $(\mathrm{E}[se_{sim}] - se_{true})/se_{true}$, where se_{true} is the true standard error as calculated based on the $5,000$ samples, and $\mathrm{E}[se_{sim}]$ is the average bootstrap standard error estimate based on all $5,000$ bootstrap replications.

All of the calculations were conducted using the freely available statistical software R and the Matrix package;[2,3] this is also the case for the case studies presented in the next section. All code is available online. The figures were created using tikz.[4]

Table 1: Results of the bootstrap simulation study; relative bias by method, sample size, and data-generating process

	Multinomial bootstrap					Block bootstrap				
Sample size	50	100	250	500	1000	50	100	250	500	1000
	i.i.d. data-generating process									
se(n_1)	0.11	0.04	0.00	-0.02	-0.01	0.07	0.03	0.01	0.00	0.00
se(e_1)	0.09	0.03	0.00	-0.01	-0.01	0.07	0.04	0.01	0.01	0.01
	non-i.i.d. data-generating process (1)									
se(n_1)	0.19	0.02	-0.04	-0.07	-0.06	0.19	0.07	0.03	0.00	0.00
se(e_1)	0.22	0.03	0.00	-0.03	-0.03	0.20	0.05	0.03	0.00	0.00
	non-i.i.d. data-generating process (2)									
se(n_1)	0.24	-0.08	-0.13	-0.15	-0.15	0.22	0.05	0.02	0.00	0.00
se(e_1)	0.31	0.12	0.09	0.09	0.08	0.17	0.03	0.01	0.01	0.01

3 Bootstrap simulation results

Results of our simulations are shown in Table 1. The upper part of the table shows results for the population created using the i.i.d. data-generating process, whereas the middle and the lower part give results for the populations with unobserved heterogeneity. For instance, when the block bootstrap is applied to the i.i.d. data-generating process and the sample size is $1,00$, then on average the standard error estimated by the block bootstrap is between 3% (se(e_1)) and 4% (se(n_1)) higher than the true value.

Relative bias differs considerably by sample size and data generating process. In case of i.i.d. sampling relative bias is mostly negligible, except for very small samples, as can be seen in the upper part of the table; the model-based and the block bootstrap perform equally well. When there is unobserved heterogeneity, but only to a small degree, as is shown in the middle part of the table, the model-based and the block bootstrap again perform well for large samples; but for very small sample size of 50 individuals relative bias can be considerable for both bootstrap methods. In case of strong heterogeneity (lower part of the table), the two bootstrap methods differ more: If sample size is small relative bias again is observed to be sizable, but it decreases more slowly with sample size for the model-based method than for the block bootstrap. For the latter relative bias is mostly small, whereas for the model-based bootstrap it can be high even for moderate-sized and large samples.

Overall, the block bootstrap seems to perform better when the i.i.d. assumption is violated. If the data generating process is i.i.d., both methods perform well. Irrespective of the bootstrap method and the data generating process small sample size seems problematic,

and both the model-based and the block bootstrap will overestimate the true sampling variation.

References

[1] Frishman F. On the arithmetic means and variances of products and ratios of random variables. In *Statistical Distributions in Scientific Work*. Reidel, 1975. pp. 401–406.

[2] R Core Team. R: A language and environment for statistical computing, 2015. Vienna, Austria.

[3] Bates D and Maechler M. Matrix: Sparse and dense matrix classes and methods, 2016. R package version 1.2-4.

[4] Tantau T. The TikZ and PGF packages, 2013. Manual for version 3.0.0.
